# Different Duration of Prone Positioning Treatment for Patients with Acute Respiratory Distress Syndrome in Intensive Care Unit Patients: A Prospective Randomized Clinical Study

**DOI:** 10.3390/jcm14207261

**Published:** 2025-10-14

**Authors:** Chien-Wei Hsu, Shan-Mei Liu, Chin-Yao Yang, Shu-Fen Sun, Shu-Hung Kuo, Kao-An Chu

**Affiliations:** 1 Department of Intensive Care Unit, Kaohsiung Veterans General Hospital, 386, Ta-Chung 1st Road, Kaohsiung 813779, Taiwan; peacefulgray@vghks.gov.tw; 2 Department of Medicine, School of Medicine, National Yang Ming Chiao Tung University, 155, Section 2, Linong Street, Beitou District, Taipei 112304, Taiwan; sfsun@vghks.gov.tw; 3 Department of Chest Medicine, Kaohsiung Veterans General Hospital, 386, Ta-Chung 1st Road, Kaohsiung 813779, Taiwan; smliu630@vghks.gov.tw (S.-M.L.); ycyaoyao@vghks.gov.tw (C.-Y.Y.); kachu@vghks.gov.tw (K.-A.C.)

**Keywords:** acute respiratory distress syndrome, driving pressure, lung protective strategies, oxygenation, prone position

## Abstract

**Introduction:** Prolonged prone positioning, exceeding 16 h, has been associated with reduced mortality among patients with moderate to severe acute respiratory distress syndrome (ARDS). Extending the duration of prone positioning may provide greater therapeutic benefits. This study aims to assess the clinical outcomes between 16 h and 24 h prone positioning therapy in patients with moderate to severe ARDS. **Methods:** This prospective randomized clinical trial was conducted in the intensive care unit of a university-affiliated tertiary medical center. Patients were randomly assigned to receive either 16 h or 24 h prone positioning therapy. All participants were managed according to a standardized protocol incorporating low tidal volume and protective lung strategies. **Results:** Out of 45 patients diagnosed with moderate to severe ARDS requiring mechanical ventilation, 21 were allocated to the 16 h prone positioning group and 24 were assigned to the 24 h group. There were no significant differences in PaO_2_/FiO_2_ ratios, driving pressure, or serum lactate levels between the two groups. The first session of prone positioning resulted in significantly greater PaO_2_/FiO_2_ improvement compared to the second session. The 24 h group showed a trend toward requiring fewer prone positioning sessions than the 16 h group. Secondary outcomes did not differ significantly between groups. **Conclusions:** Both 16 h and 24 h prone positioning therapies improved oxygenation in patients with moderate to severe ARDS. The 24 h prone group showed a trend toward fewer sessions, potentially reducing clinical workload. The first prone session provided greater oxygenation improvement compared to the second session.

## 1. Introduction

Acute respiratory distress syndrome (ARDS) is a life-threatening condition, and most patients require mechanical ventilation. Treatment options include low tidal volume ventilation, corticosteroids, extracorporeal membrane oxygenation (ECMO), and prone positioning [1]. Prone positioning helps recruit and stabilize dependent lung regions [2], thereby improving oxygenation and reducing mortality in moderate-to-severe ARDS cases. Current guidelines recommend at least 16 h of prone positioning therapy [3]. Yan Y et al. reported that prolonged prone positioning (≥16 h) is associated with reduced 28-day mortality and improved response rates without increasing complications [4]. Other studies have suggested that longer durations may provide greater benefit [5,6]. However, Hochberg CH et al. showed no significant effect of extended (≥24 h) versus standard (16–24 h) prone duration on mortality, ventilator liberation, or ICU discharge for coronavirus disease (COVID-19)-associated ARDS [7]. The optimal duration of prone positioning remains uncertain. In light of this, this study was designed to compare clinical outcomes between 16 h and 24 h prone positioning in patients with moderate-to-severe ARDS.

## 2. Materials and Methods

### 2.1. Enrollment

The study was conducted over three years (July 2020 to July 2023) in an adult intensive care unit (ICU). Eligible participants were ≥20 years old, diagnosed with moderate-to-severe ARDS (PaO_2_/FiO_2_ ratio < 150 mmHg, positive end-expiratory pressure (PEEP) ≥ 5 cmH_2_O, FiO_2_ > 60%). They were managed under protective lung ventilation protocols (tidal volume 4–8 mL/kg, plateau pressure ≤ 30 cmH_2_O). Patients were required to have an expected survival of at least 24 h. Exclusion criteria included abdominal surgery with an open wound, massive hemoptysis, intracranial hemorrhage, pregnancy, and spine or pelvic fractures. This study was approved by the Institutional Review Board of Kaohsiung Veterans General Hospital (VGHKS19-CT11-14, approval date: 20 November 2019). Before enrolling the first patient, the study was registered at clinicaltrials.gov (NCT04391387, registration date: 20 March 2020). This study was conducted in accordance with the Declaration of Helsinki. No conflicts of interest were declared.

### 2.2. Randomization

**Figure 1:**
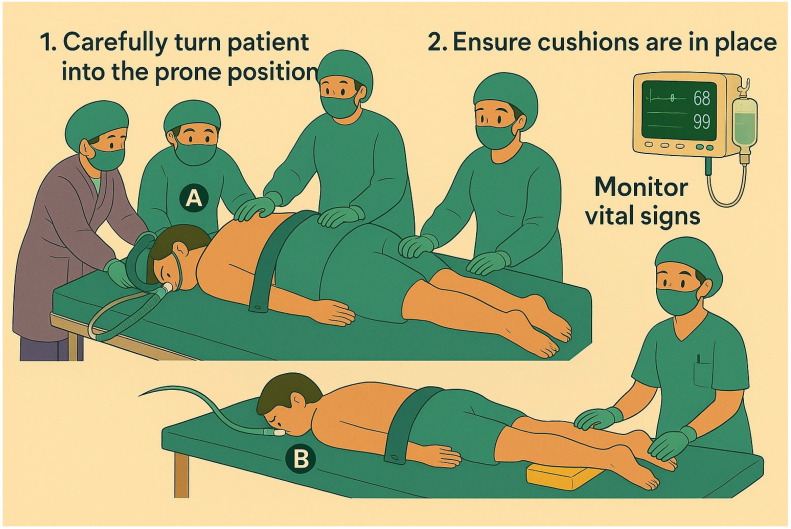
The diagram of prone positioning treatment. A: Turn patient to the prone position and avoid tube dislodgement; B: Ensure cushion is in place and monitor vital signs.

After obtaining informed consent from the patients or their next of kin, participants were randomized 1:1 using a computer-generated sequence. Allocation was concealed in sealed containers. Caregivers were blinded to the randomization sequence. Upon assignment, baseline arterial blood gas, serum lactate, and driving pressure measurements were recorded before positioning. Prone positioning was performed after sedation and opioid administration (Figure 1).

Following prone positioning, arterial blood gas, driving pressure, and serum lactate levels were measured at the first, 8th, 16th, and 24th hours. These parameters were re-evaluated 8 h after discontinuation of prone positioning. If patients achieved a PaO_2_/FiO_2_ ratio ≥ 150 mmHg on FiO_2_ ≤ 0.6 and PEEP ≤ 10 cmH_2_O at least 4 h in the supine position, the next session of prone positioning therapy was discontinued [8]. Conversely, if patients did not meet these criteria, the next session of prone positioning therapy was initiated, with subsequent arterial blood gas, driving pressure, and serum lactate checks.

Demographic data collected at randomization included primary ICU admission diagnosis, age, gender, body mass index (BMI), number of organ failures, Acute Physiology and Chronic Health Evaluation (APACHE) II score, serum lactate and arterial blood gas levels, days between intubation and prone positioning, days between ARDS onset and prone positioning, and pulmonary or extra-pulmonary causes of ARDS.

Furthermore, specific medications administered during the study period, such as steroids (methylprednisolone, hydrocortisone, dexamethasone, prednisolone), antibiotics, sedatives, muscle relaxants, and opioids, were recorded.

The protective lung strategy for ventilator settings entailed maintaining a tidal volume of 4–8 mL per predicted body weight, with plateau pressure kept at ≤30 cmH_2_O [9].

### 2.3. Observations

Patients were monitored daily for the occurrence of pressure sores, endotracheal tube obstruction, tube dislodgement, ventilator-associated pneumonia (VAP), and the need for rescue use of ECMO. Monitoring of patients continued throughout the duration of prone positioning therapy, extending until the cessation of the final session.

### 2.4. Definitions

The diagnosis of ARDS follows the Berlin definition, as shown in Table 1 [10]:

Driving pressure is calculated as the difference between plateau pressure and PEEP, with plateau pressure defined as the pressure after the inspiratory pause.

A positive response to prone positioning therapy is indicated by an increase in the PaO_2_/FiO_2_ ratio of ≥20% following prone positioning. Mortality is defined as death at hospital discharge.

Diagnosis of VAP required the agreement of two pulmonologists. All radiographs were reviewed independently by each pulmonologist, without knowledge of clinical details. The diagnosis followed a modified version of the criteria of the National Nosocomial Infection Surveillance (NNIS) system developed by the Centers for Disease Control [11].

### 2.5. Outcomes

The primary outcomes measured in this study were the changes in the PaO_2_/FiO_2_ ratio following each prone positioning session, variations in driving pressure, and serum lactate levels. Secondary outcomes included the number of prone positioning sessions required, lengths of ICU and hospital stays, duration of mechanical ventilation, occurrences of pressure sores, tube dislodgement, endotracheal tube obstruction, incidents of ventilator-associated pneumonia, and mortality.

### 2.6. Statistical Analysis

The statistical analysis was performed using SPSS version 29.0 (SPSS, Inc., Chicago, IL, USA). A comparison between the two treatment groups was conducted based on the intent-to-treat principle. Data were presented as mean ± standard deviation, percentage, or median with interquartile ranges (IQRs). Student’s *t*-test was employed for comparing continuous variables with a normal distribution, while the Mann–Whitney U test was utilized for continuous variables with a non-normal distribution. Dichotomous variables were compared using either the Chi-square test or Fisher’s exact test, depending on the expected frequency of occurrence. Differences in primary outcomes were analyzed using two-way analysis of variance (ANOVA), followed by post hoc analysis with Bonferroni correction. Difference of PaO_2_/FiO_2_ ratio change among different sessions of prone positioning was analyzed using two-way ANOVA. All *p*-values were two-tailed, and statistical significance was considered at *p* < 0.05.

## 3. Results

### 3.1. Demographics

**Figure 2:**
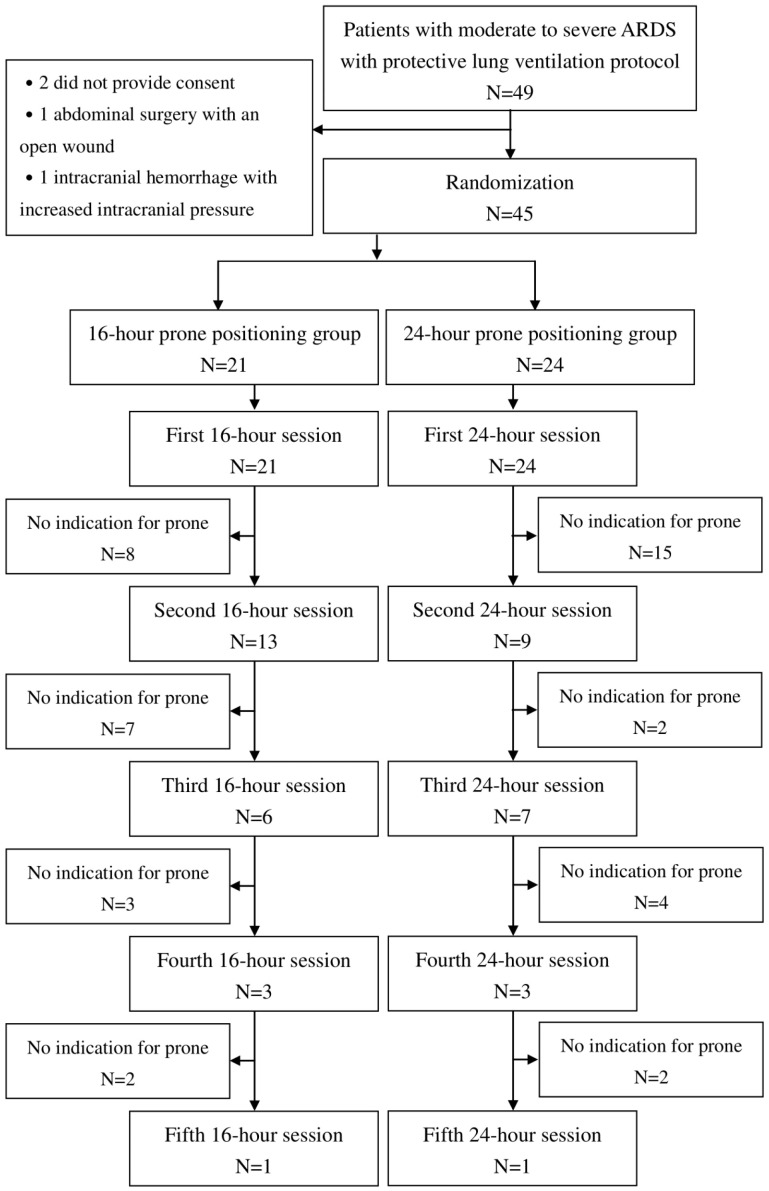
The study flow chart.

Forty-nine patients initially meeting the inclusion criteria were enrolled in the study. However, two patients did not provide consent, one had undergone abdominal surgery with an open wound, and another had an intracranial hemorrhage with increased intracranial pressure. Consequently, 45 patients were included, with 21 receiving 16 h of prone positioning therapy and 24 receiving 24 h of therapy. The flow chart for all patients is shown in Figure 2. All patients completed the study. The study, which included six COVID-19-associated ARDS cases, ended upon reaching the investigation and research expiration date. Data were analyzed according to the intent-to-treat principle.

At randomization, demographic characteristics such as gender; age; BMI; number of organ failures; APACHE II score; use of sedation, muscle relaxants, vasopressors, or steroids; interval between intubation and prone position, time from ARDS onset to prone positioning therapy, pulmonary or extra-pulmonary cause of ARDS, and serum lactate levels were similar between the two groups (Table 2).

No statistical differences were observed in respiratory parameters before the study period (Table 3), indicating homogeneity between the two groups.

### 3.2. Primary Endpoints

**Figure 3:**
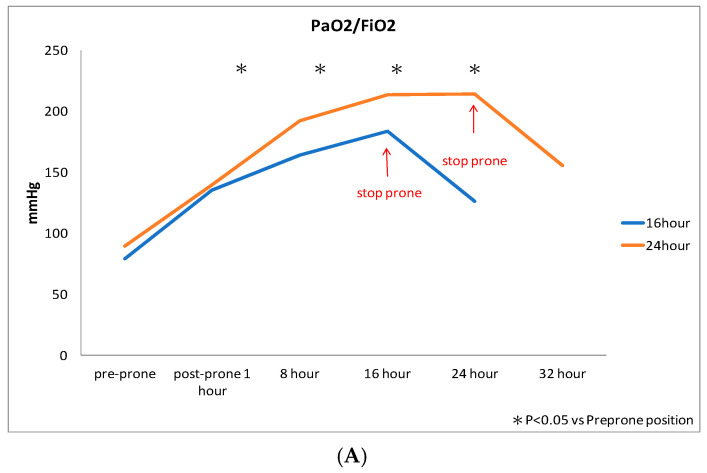

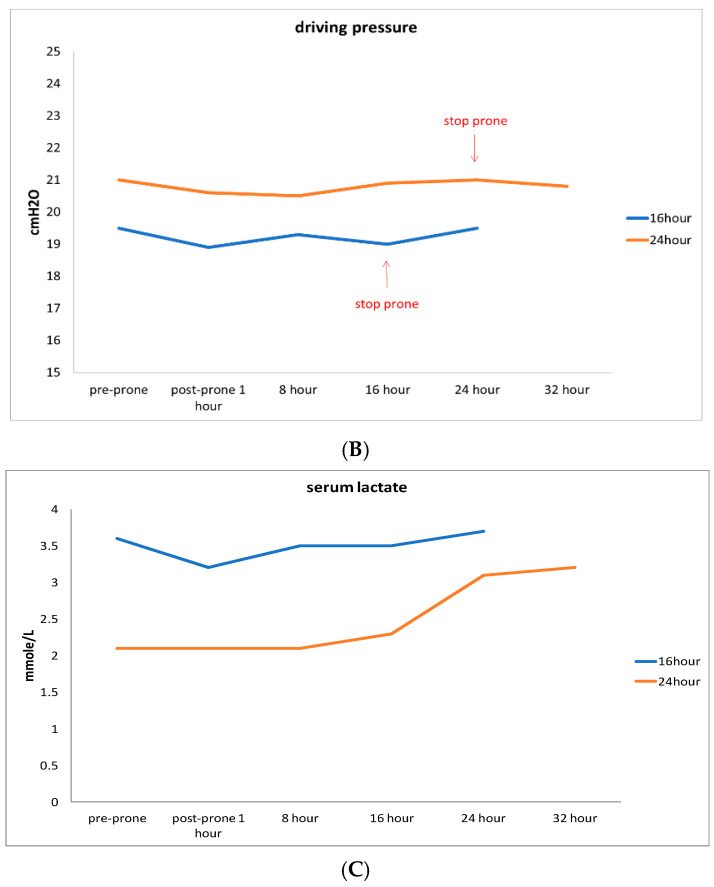
(**A**) Changes in PaO_2_/FiO_2_ from pre-prone positioning to post-prone positioning. *: *p* < 0.05 indicates a significant difference between post-prone positioning and pre-prone positioning in the 24 h and 16 h groups. (**B**) Changes in driving pressure from pre-prone positioning to post-prone positioning. (**C**) Changes in serum lactate from pre-prone positioning to post-prone positioning.

Both groups demonstrated significant improvement in PaO_2_/FiO_2_ after prone positioning (Figure 3A), while there was no significant change in driving pressure (Figure 3B) or serum lactate levels (Figure 3C) during prone positioning. Notably, PaO_2_/FiO_2_ significantly increased within the first hour of prone positioning, but often decreased upon transitioning from prone to supine position (Figure 3A). There were no significant differences in PaO_2_/FiO_2_ driving pressure or serum lactate levels between the 16 h group and the 24 h group (Figure 3A–C).

**Figure 4:**
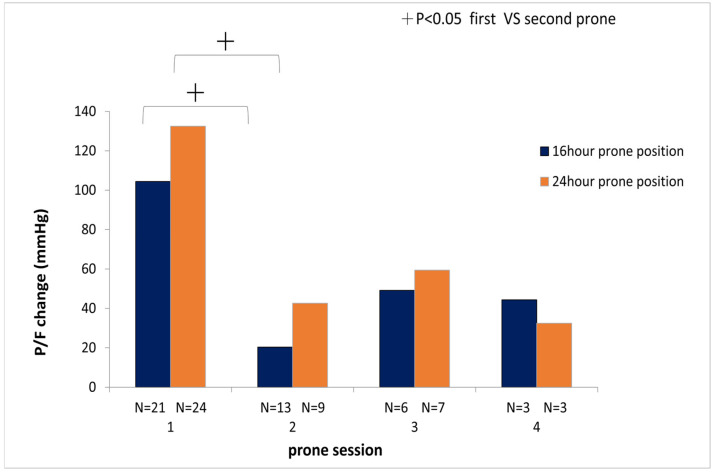
Changes in PaO_2_/FiO_2_ between the 16 h and 24 h groups were evaluated during the first, second, third, and fourth sessions of prone positioning therapy. +: *p* < 0.05 indicates a significant difference in PaO_2_/FiO_2_ change between the first and second sessions of prone positioning therapy.

Additionally, a decreasing trend was observed in the change of PaO_2_/FiO_2_ between pre- and post-prone positioning. With subsequent sessions, there was a significantly greater change in the PaO_2_/FiO_2_ ratio in the first session compared to the second session of prone positioning (24 h group: first session 135 ± 112.8 mmHg vs. second session 42.6 ± 50.5 mmHg, *p* < 0.05; 16 h group first session 104.4 ± 84.9 mmHg vs. second session 20.4 ± 91.6 mmHg, *p* < 0.05) (Figure 4).

### 3.3. Secondary Clinical Outcomes

Secondary clinical outcomes, including the number of prone positioning sessions, changes in PaO_2_/FiO_2_ after discontinuation of prone positioning, incidence of tube dislodgement, endotracheal tube obstruction, pressure sores, ICU days, ventilator days, hospital days, and occurrences of VAP are summarized in Table 4. Patients undergoing 24 h prone positioning therapy exhibited a trend toward a lower rate of repeated prone positioning sessions compared to those receiving 16 h therapy (37.5% vs. 61.9%, *p* = 0.06) (Table 4), although this difference did not reach statistical significance. No significant differences in complications such as pressure sores, tube dislodgement, endotracheal tube obstruction, or VAP were observed between the two groups. The mortality was not significantly different between the 16 h group (57.1%) and the 24 h group (54.2%), with both groups showing a high responder rate (95.2% vs. 95.8%). Additionally, there was no significant difference in the need for rescue ECMO use. The 30-day outcomes, including the length of ICU-free days, ventilator-free days, and survival while liberated from the ventilator, did not exhibit any significant differences (Table 4).

## 4. Discussion

This study revealed that prone positioning therapy effectively enhanced oxygenation for patients with moderate-to-severe ARDS in both the 16 h and 24 h prone positioning groups. An early session of prone positioning had a greater PaO_2_/FiO_2_ ratio improvement. However, it did not significantly improve driving pressure or serum lactate levels. The 24 h duration of prone positioning demonstrated a tendency toward requiring fewer therapy sessions compared to the 16 h duration.

In previous studies, the increase in PaO_2_ ranged from 23 to 78 mmHg, with PaO_2_/FiO_2_ improving by 21 to 161 mmHg [12]. Our study shows a PaO_2_/FiO_2_ increase of 114.1 mmHg. The improvement results from a reduction in shunt and ventilation-perfusion heterogeneity that occurs because the lungs, which anatomically resemble a cone, fit into their cylinder-like thorax enclosure with less distortion when patients are prone versus supine [13,14].

Driving pressure, which reflects global lung strain [15,16], is recognized as a risk factor for ARDS in mechanically ventilated patients [17]. Reductions in driving pressure have been strongly linked to improved survival [15]. However, our study did not show that driving pressure was reduced significantly during prone positioning. This aligns with prior findings that driving pressure may not significantly change during prone positioning in ARDS patients [18]. While prone positioning for more than 12 h reduces mortality in patients with moderate-to-severe ARDS [19], this effect is not directly related to changes in driving pressure. In patients with ARDS, the proportion of lung available for ventilation is markedly reduced, and respiratory system compliance is directly related to functional lung size. Adjusting tidal volume according to a patient’s respiratory system compliance correlates directly with transpulmonary pressure and is associated with improved survival in ARDS [15].

Although prone positioning significantly improves oxygenation, it does not substantially affect serum lactate levels. Lactate is influenced by multiple factors, including underlying diseases, medications, cellular metabolism, tissue perfusion, and regional ischemia [20]. A decrease in lactate reflects both improved microcirculation and enhanced lactate clearance, rather than simply increased oxygen delivery. Consistent with our results, Yoshida T et al. reported no significant reduction in serum lactate between supine and prone positioning (supine: 15 mg/dL vs. prone: 13 mg/dL) [21].

Importantly, the initial prone session produced a significantly greater improvement in PaO_2_/FiO_2_ compared to subsequent sessions. This suggests that prone positioning may be effective when initiated early (<3 days) during the exudative phase, when congestive and compressive atelectasis are predominant, compared with the later phase of ARDS (>1 week) [22].

Most observational studies have reported no significant improvement in the partial pressure of carbon dioxide (PaCO_2_) with prone positioning [12], consistent with our findings. However, clinical outcomes tend to be more favorable when prone positioning results in a reduced PaCO_2_ at the same minute ventilation [23].

Our study also found that patients in the 16 h group tended to require more repeated prone sessions. This was often due to worsening oxygenation after discontinuation, with PaO_2_/FiO_2_ falling below 150 mmHg, necessitating resumption of prone positioning therapy. In contrast, 24 h prone positioning therapy provided more sustained oxygenation improvement, reducing the need for repeated sessions and thereby lowering clinical workload.

The overall response rate to prone positioning therapy in our cohort was 95.5%. Lee et al. reported that responders had a significantly shorter time from ARDS onset to prone ventilation (8.4 ± 2.9 vs. 15.2 ± 5.7 days, *p* < 0.05), with a response rate of 63.6% in that study [24]. In our study, the average interval from ARDS onset to the first prone positioning was one day. Similarly, L’Her E et al. demonstrated a 96% response rate when prone therapy was initiated within the first 24 h [25].

Prone positioning is associated with a higher risk of pressure sores compared with the supine position, particularly in patients over 60 years of age or with a BMI above 28.4 [26]. Additionally, endotracheal tube obstruction is another common complication associated with prone positioning. Lee JM et al. demonstrated that prone positioning increased the risk of endotracheal tube obstruction 2.16 fold (95% CI, 1.53–3.05, *p* < 0.001), based on a previous meta-analysis of 11 randomized controlled studies [27]. However, there was no significant difference in complications between the 16 h and 24 h groups, consistent with recent findings by Page DB et al. [28].

This study has several limitations. The small sample size may limit the power to detect differences between groups. Based on P/F outcomes, a sample size of 794 (397 per group) would be required to achieve 80% power at α = 0.05. Furthermore, this was a single-center, non-double-blinded randomized controlled trial. Larger multicenter studies are warranted to confirm these findings.

## 5. Conclusions

Prone positioning significantly enhances oxygenation in patients with moderate to severe ARDS within the first hour of therapy. Early initiation of prone positioning yields greater oxygenation benefits. The 24 h prone positioning group tended to require fewer sessions than those in the 16 h group. However, there were no significant differences in oxygenation, driving pressure, serum lactate levels, mortality, or complications.

## Figures and Tables

**Table 1:** Acute respiratory distress syndrome.

Timing	Within I week of a known clinical insult or new/worsening respiratory symptoms		
Chest imaging (Chest X-ray or CT scan)	Bilateral opacities—not fully explained by effusions, lobar/lung collapse, or nodules		
Origin of Edema	Respiratory failure not fully explained by cardiac failure or fluid overload; Need objective assessment (e.g., echocardiography)to exclude hydrostatic edema if no risk factor present		
	Mild	Moderate	Severe
Oxygenation	200 < PaO_2_/FiO_2_ ≤ 300 with PEEP or CPAP ≥ 5 cmH_2_O	100 < PaO_2_/FiO_2_ ≤ 200 with PEEP ≥ 5 cmH_2_O	PaO_2_/FiO_2_ ≤ 100 with PEEP ≥ 5 cmH_2_O

Definitions of abbreviations: CT, computed tomography; PaO_2_, partial pressure of oxygen; FiO_2_, fraction of inspired oxygen; CPAP, continuous positive airway pressure.

**Table 2:** The demographic characteristics of patients in the 16 h group and the 24 h group before the initiation of prone positioning therapy.

Characteristic	16 h (N = 21)	24 h (N = 24)	*p*-Value
Gender (M/F)	11/10	10/14	0.47
Age, year	71.1± 13.6	69.0 ± 11.2	0.59
BMI (kg/m^2^)	23.2 ± 3.3	24.9 ± 6.3	0.27
Organ failure number	2.3 ± 1.2	2.3 ± 1.1	1.00
APACHE II score Invasive mechanical ventilation (%)	26.6 ± 7.3 21 (100)	27.4 ± 7.7 24 (100%)	0.72 0.99
Sedation (%)	21 (100)	24 (100)	0.99
Muscle relaxant (%)	18 (85.7)	24 (100)	0.06
Vasopressor (%)	18 (85.7)	17 (70.8)	0.22
Steroid (%) Intubation to prone day (median, IQR)	17 (81.0) 2 (1–3)	20 (83.3) 2 (1–3)	0.81 0.36
ARDS to prone day	1.1 ± 1.9	1.0 ± 1.3	0.91
Pulmonary ARDS (%)	16 (76.2)	20 (83.3)	0.55
Extrapulmonary ARDS (%)	5 (23.8)	4 (16.7)	0.55
Serum lactate (mmole/L)	3.6 ± 3.5	2.1 ± 1.1	0.08

Definition of abbreviations: M/F, male/female; BMI, body mass index; APACHE, Acute Physiologic and Chronic Health Evaluation; IQR, interquartile range; ARDS, acute respiratory distress syndrome.

**Table 3:** The disparity in ventilator parameters between the 16 h group and the 24 h group before the initiation of prone positioning therapy.

Characteristic	16 h (N = 21)	24 h (N = 24)	*p*-Value
PaO_2_/FiO_2_ (mmHg)	79.3 ± 31.9	89.7 ± 30.5	0.27
PaO_2_ (mmHg)	75.4 ± 28.9	78.9 ± 23.4	0.67
PaCO_2_ (mmHg)	46.3 ± 28.9	53.3 ± 18.5	0.12
pH	7.4 ± 0.1	7.3 ± 0.1	0.13
Respiratory rate (breath/minute)	23.1 ± 4.9	22.1 ± 5.8	0.55
Tidal volume (mL)	456.4 ± 120.4	462.8 ± 117.2	0.86
PEEP (cmH_2_O)	12.0 ± 3.1	11.5 ± 2.6	0.53
Compliance (mL/cmH_2_O)	23.8 ± 8.9	23.2 ± 8.8	0.82
Driving pressure (cmH_2_O)	19.5 ± 4.5	21.0 ± 4.2	0.25
Mean airway pressure (mmHg)	19.2 ± 3.1	19.3 ± 3.6	0.92

Definition of abbreviations: PaO_2_, partial pressure of oxygen; FiO_2_, fraction of inspired oxygen; PaCO_2_, partial pressure of carbon dioxide; PEEP, positive end expiratory pressure.

**Table 4:** The variation in clinical outcomes between the 16 h group and the 24 h group following prone positioning.

Characteristic	16 h (N = 21)	24 h (N = 24)	*p*-Value
Session > 1 of prone positioning (%) Total prone positioning session per patient (median, IQR)	13 (61.9) 2 (1–3)	9 (37.5) 1 (1–3)	0.06 0.77
Change of P/F from prone to supine position (mmHg)	−111.4 ± 134.7	−74.8 ± 71.9	0.28
Tube dislodgement (%)	1 (4.8)	0 (0)	0.28
Endotracheal tube obstruction (%)	1 (4.8)	2 (8.3)	0.63
Pressure sore (%)	1 (4.8)	4 (16.7)	0.13
Ventilator-associated pneumonia (%)	1 (4.8)	3 (12.5)	0.35
Weaning ventilator (%)	9 (42.9)	9 (37.5)	0.71
Mortality at hospital discharge (%)	12 (57.1)	13 (54.2)	0.86
Change in PaCO_2_ in the first session (mmHg)	3.2 ± 10.9	9.6 ± 15.3	0.12
Change in P/F after prone positioning in the first session (mmHg)	104.4 ± 84.9	123.1 ± 105.2	0.52
PaO_2_/FiO_2_ responder (%)	20 (95.2)	23 (95.8)	0.88
Rescue ECMO (%)	0 (0)	0 (0)	0.99
30-day outcomes			
ICU-free days	18.2 ± 7.7	14.4 ± 7.2	0.28
Ventilator-free days	16.1 ± 9.1	9.7 ± 7.3	0.16
Alive and liberated from ventilator (%)	8 (38.1)	7 (29.2)	0.53

Definition of abbreviations: IQR, interquartile range; P/F, PaO_2_/FiO_2_; ICU, intensive care unit; PaCO_2_, partial pressure of carbon dioxide; PaO_2_, partial pressure of oxygen; FiO_2_, fraction of inspired oxygen; ECMO, extracorporeal membrane oxygenation.

## Data Availability

The data presented in this study are available on request from the corresponding author. This author takes responsibility for all aspects of the reliability and freedom from bias of the data presented and their discussed interpretations.
